# The association between skeletal muscle mass and all-cause mortality in acute exacerbation of chronic obstructive pulmonary disease

**DOI:** 10.3389/fnut.2025.1568527

**Published:** 2025-04-08

**Authors:** Tianye Li, Hao Xu, Lefu Chen, Yixin Xu, Yanhong Zheng, Hongjun Zhao, Chengshui Chen, Zaisheng Zhu

**Affiliations:** ^1^Department of Pulmonary and Critical Care Medicine, Key Laboratory of Interventional Pulmonology of Zhejiang Province, The First Affiliated Hospital of Wenzhou Medical University, Wenzhou, China; ^2^Department of Pulmonary and Critical Care Medicine, Clinical Research and Trial Center, Zhejiang Province Engineering Research Center for Endoscope Instruments and Technology Development, Quzhou People’s Hospital, The Quzhou Affiliated Hospital of Wenzhou Medical University, Quzhou, China; ^3^Department of Internal Medicine, Nassau University Medical Center, East Meadow, NY, United States; ^4^Department of General Medicine, Department of Pulmonary and Critical Care Medicine, Key Laboratory of Interventional Pulmonology of Zhejiang Province, The First Affiliated Hospital of Wenzhou Medical University, Wenzhou, China

**Keywords:** chronic obstructive pulmonary disease, acute exacerbation, skeletal muscle mass, skeletal muscle index, all-cause mortality

## Abstract

**Background:**

Acute exacerbation of chronic obstructive pulmonary disease (AECOPD) significantly impacts patient quality of life and prognosis. Skeletal muscle mass loss, a systemic manifestation of COPD, has garnered increasing attention, but its association with all-cause mortality in AECOPD remains unclear. This study aimed to quantitatively assess skeletal muscle mass in AECOPD patients using computed tomography and explore the association between muscle mass-related indices and all-cause mortality risk.

**Methods:**

A total of 319 patients were enrolled in this single-center retrospective cohort study. Muscle mass-related indices, including skeletal muscle area, mean muscle density, intermuscular fat density, intermuscular fat area, and skeletal muscle index (SMI), were considered as independent variables. All-cause mortality was considered as the dependent variable. Univariate and multivariate Cox regression, subgroup and sensitivity analyses, receiver operating characteristic curve (ROC), restricted cubic spline plot (RCS), and Kaplan–Meier survival curves were used to examine the association between these indices and all-cause mortality in AECOPD patients.

**Results:**

During a median follow-up of 14.63 (6.33, 21.13) months, the all-cause mortality was 113 (35.4%). Multivariate Cox regression revealed that, regardless of whether SMI was grouped based on the median of 26.08 or the cut-off point of 24.01, the low SMI group had a higher risk of all-cause mortality (HR: 0.495, 95% CI: 0.330–0.743, *p* = 0.001; HR: 0.400, 95% CI: 0.270–0.592, *p* < 0.001). Moreover, as a continuous variable, lower levels of SMI were independently associated with a higher risk of all-cause mortality (SMI, HR = 0.964, 95% CI: 0.934–0.996, *p* = 0.027; Standardized SMI, HR = 0.748, 95% CI: 0.578–0.967, *p* = 0.027). Subgroup and sensitivity analyses confirmed the significant association between SMI and all-cause mortality (*p* < 0.05). ROC analysis showed good predictive value for SMI (area under the curve = 0.663, 95% CI: 0.559–0.728, *p* < 0.001), and RCS analysis revealed a non-linear relationship between SMI and mortality (*p* nonlinear = 0.019). The Kaplan–Meier survival curves analysis indicated that regardless of whether SMI was grouped by median or by cut-off point, there were significant differences in the survival probability of all-cause mortality among different SMI groups, with the low SMI group having a poorer prognosis (*p* < 0.001).

**Conclusion:**

Among patients with AECOPD, higher levels of SMI are significantly associated with a lower risk of all-cause mortality, suggesting that SMI may have important prognostic value in the assessment of mortality risk in AECOPD patients.

## 1 Introduction

Chronic Obstructive Pulmonary Disease (COPD) is a chronic inflammatory disease characterized by persistent airflow limitation, typically presenting with a progressive course of exacerbations and significant systemic effects (1, 2). Acute exacerbation (AE) is a key event in the natural course of COPD and is strongly associated with reduced quality of life, increased hospitalization, and higher mortality in patients (1, 3). However, AECOPD is not only an important driver of poor prognosis in patients with COPD but also further promotes muscle loss through systemic inflammation (4–6). Muscle loss not only further reduces the quality of life and motor function in COPD patients but also increases the health burden of patients (7, 8). Therefore, it is crucial to assess muscle loss in patients with AECOPD and its impact on their poor prognosis (9, 10).

In recent years, the reduction of skeletal muscle mass in patients with COPD – characterized by the loss of skeletal muscle mass and function – has received increasing attention as a systemic manifestation, and the pathological mechanism is mainly related to chronic inflammation, long-term hypoxemia, malnutrition, and lack of physical activity (11–13). Several studies have shown that reduced muscle mass significantly impairs exercise capacity and quality of life in COPD patients and is strongly associated with an increased risk of acute exacerbations and adverse outcomes (10, 14). Furthermore, reduced muscle mass not only worsens dyspnea, but also impairs patients’ ability to cope with acute stress (e.g., acute exacerbations), which can lead to increased hospitalization rates and a higher risk of death (15, 16). This suggests that muscle mass loss and its related indicators may have important clinical utility in assessing the severity and poor prognosis of patients with AECOPD.

With the development of imaging techniques, particularly CT-based skeletal muscle assessment, a reliable and objective method has been established for assessing muscle mass loss and quantifying muscle mass and function (17–20). At present, CT-derived parameters, including skeletal muscle area, mean muscle density, mean intermuscular fat density, intermuscular fat (IMAT), muscle cross-sectional area (CSA) and skeletal muscle index (SMl), have been widely considered to be accurate indicators for evaluating muscle mass loss, muscle mass and fat infiltration (11, 21). For example, studies have shown that the cross-sectional area and density of the pectoralis major muscle are significantly correlated with the severity of airflow limitation, lung function decline, and quality of life in COPD patients, which indicates that these indicators have certain application value in the evaluation of COPD patients (22–24). Other studies have also shown that skeletal muscle fatty infiltration is strongly associated with increased mortality in critically ill patients, such as those with sepsis or severe community-acquired pneumonia, highlighting its importance in the management of critically ill patients (25, 26). However, these muscle mass related indicators have not been systematically explored in the prognostic assessment of AECOPD patients.

Therefore, based on the above research background, this study aimed to quantitatively assess skeletal muscle mass in AECOPD patients using CT imaging and to investigate the association between muscle mass-related indicators and all-cause mortality in patients. By exploring the impact of muscle mass on all-cause mortality in AECOPD patients, it is expected to provide references and theoretical clues for the risk assessment of mortality in AECOPD patients.

## 2 Methods

### 2.1 Study population

**Figure 1:**
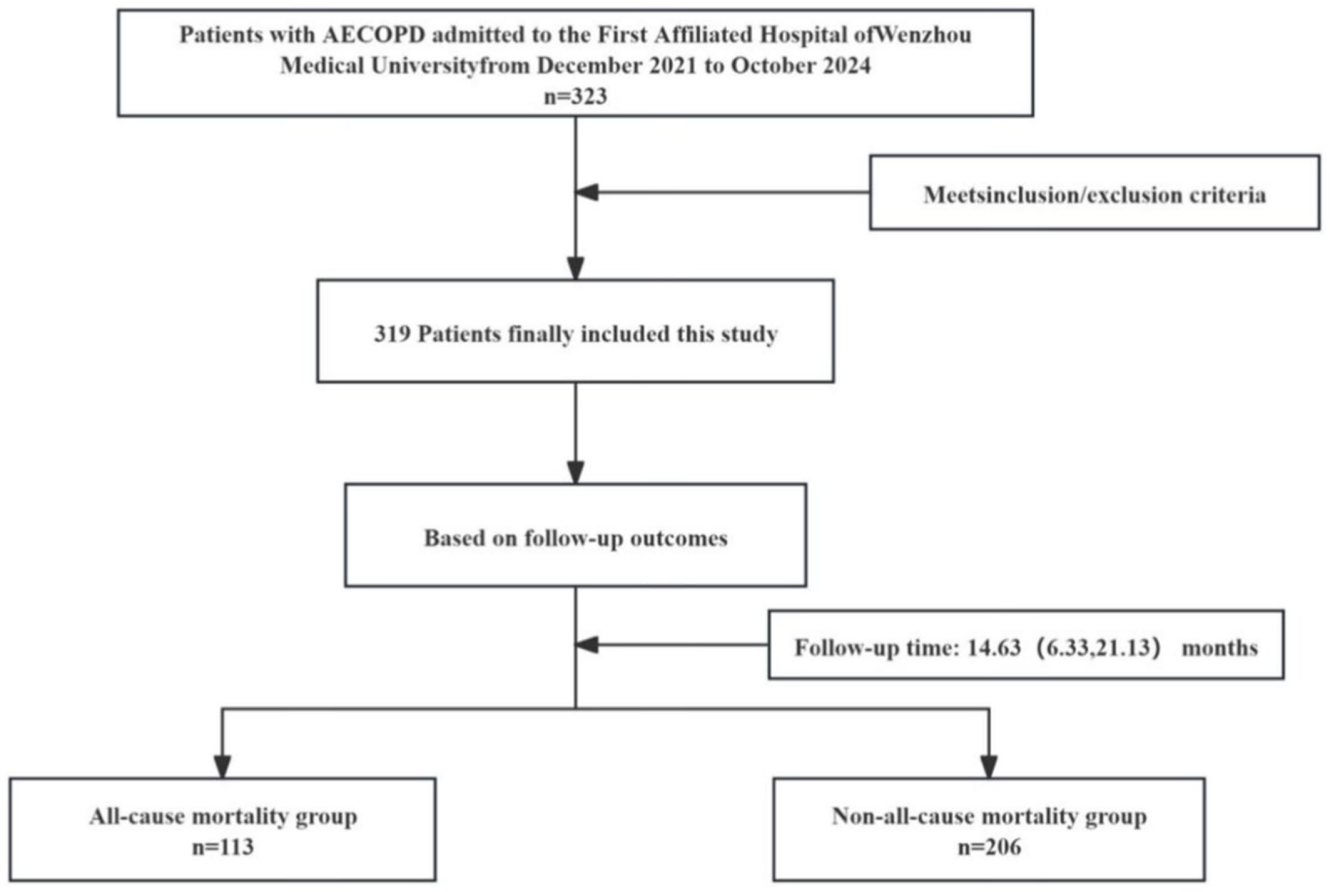
Study flow diagram. AECOPD, acute exacerbation of chronic obstructive pulmonary disease.

In this single-center, retrospective cohort study, patients with AECOPD who were hospitalized from December 2021 to October 2024 in the First Hospital of Wenzhou Medical University from China were enrolled. Inclusion criteria: (1) AECOPD met the diagnostic criteria (27); (2) age ≥ 45 years old; (3) clear consciousness, normal communication ability and consent to follow-up. Exclusion criteria: (1) patients with other lung diseases such as bronchial asthma, bronchiectasis, and lung cancer; (2) patients with mental illness or cognitive impairment; (3) patients with major organic diseases (including severe liver and kidney failure, malignant tumors); (4) patients without chest CT examination or with insufficient CT image quality to evaluate muscle mass related indicators; (5) loss of follow-up. Ultimately, 319 patients were enrolled in the study (Figure 1). This study was approved by the Medical Ethics Committee of the First Affiliated Hospital of Wenzhou Medical University (KY2023-R084), and all patients gave informed consent and volunteered to participate in this study. The study protocol was in accordance with the Declaration of Helsinki.

### 2.2 Assessment of muscle mass

This study refered to previous research methods and selected the T12 level cross-sectional CT images for evaluating muscle mass indicators (28). Each participant underwent scanning using a 16-slice spiral CT scanner (United Imaging CT, model uCT710, Shanghai United Imaging Healthcare Co., Ltd.) with a slice thickness of 5 mm and acquisition parameters of 120 kV, with mAs adjusted according to the patient’s body size. All CT images were segmented by a trained researcher who was blinded to the participants’ clinical information, and another researcher reviewed the segmented images. Muscle mass-related indicators were extracted using a software model based on the previous study (28). Muscle cross-sectional area was measured based on a muscle tissue threshold of −29 to 150 HU, including the erector spinae, latissimus dorsi, rectus abdominis, external oblique abdominis, internal oblique abdominis, and the internal and external intercostal muscles, the area of intermuscular fat (segmented based on the intermuscular tissue threshold), the radiodensity of skeletal muscle (the lower the radiodensity of skeletal muscle, the higher the degree of muscle fatty degeneration), and the radiodensity of intermuscular fat as the radiodensity of intermuscular fat tissue. SMI is defined as the muscle cross-sectional area divided by the square of height (m) (28). Two well-trained physicians also reviewed and corrected the determined regions. In order to adequately reflect the association between SMI and all-cause mortality, SMI was standardized: standardized SMI = (SMI raw value – SMI mean)/SMI standard deviation.

### 2.3 Assessment of the primary outcome

In this study, all patients were followed up from hospital discharge until death or December 2024. All patients were followed up regularly at the outpatient department and by phone after discharge, with a visit every three months. Information on mortality was obtained from the outpatient follow-up, phone follow-up and electronic medical records of readmission of the patients. All-cause mortality was considered to be the study outcome and included death from any cause. According to the occurrence of all-cause mortality, the patients were divided into two groups: non-all-cause mortality (*n* = 206) and all-cause mortality (*n* = 113).

### 2.4 Collection and definition of covariates

Data were extracted from the hospital electronic medical record system, including demographic, comorbidities, anthropometry, auxiliary examination, and blood markers. Demographic information included age, gender (male and female), smoking (yes or no) and alcohol intake (yes or no). Smoking was defined as regular smoking in the past, regardless of current abstinence. Alcohol intake was defined as regular past alcohol consumption, regardless of current alcohol consumption. Data on comorbidities included diabetes, hypertension, dyslipidemia, pulmonary heart disease, respiratory failure, and heart failure. Diabetes was defined as a previous diagnosis of diabetes or current treatment with antidiabetic drugs or a fasting plasma glucose level ≥ 7.0 mmol/L or a glycated hemoglobin level > 6.5%. Hypertension was defined as a history of previously diagnosed hypertension or systolic/diastolic blood pressure (SBP/DBP) ≥ 140/90 mmHg during the current hospitalization. Dyslipidemia was defined as total cholesterol ≥6.22 mmol/L, triglyceride ≥2.3 mmol/L, high-density lipoprotein cholesterol <1.04 mmol/L, low-density lipoprotein cholesterol ≥4.1 mmol/L, meeting any one or more of the above criteria or currently undergoing lipid-regulating drug treatment (29). Auxiliary examination data included pulmonary hypertension and pulmonary heart disease from echocardiographic, as well as PH value, fraction of inspired oxygen (FiO_2_)_,_ AECOPD severity and respiratory failure assessed by blood gas analysis. Pulmonary hypertension was graded according to the results reported by the echocardiographer. The severity of respiratory failure was classified into three groups based on blood gas analysis: mild (partial pressure of arterial carbon dioxide (PaCO2) < 50 mmHg), moderate (50 mmHg ≤ PaCO2 ≤ 60 mmHg), and severe (PaCO2 > 60 mmHg). FiO2 was defined as the inspired oxygen concentration, which is the proportion of oxygen in the inhaled gas, and is calculated as 21 + oxygen flow x 4. Respiratory failure was defined as partial pressure of arterial oxygen (PaO2) < 60 mmHg with or without PaCO2 > 50 mmHg at sea level, resting state, breathing air conditions. Duration of intubation (h) was defined as the length of time the patient was intubated via orotracheal intubation and/or tracheotomy after admission.

Biomarkers included anthropometric and blood markers, Anthropometric indicators included body mass index ‌(BMI), SBP, DBP, heart rate, and arterial oxygen saturation (‌SaO2). BMI was defined as weight (kg)/height (m^2^). Blood marker included lactate, sodium, potassium, calcium, glucose, C-reactive protein (CRP), procalcitonin (PCT), white blood cell (WBC), hemoglobin, platelet (PLT), albumin (ALB), alanine aminotransferase (ALT), aspartate aminotransferase (AST), total bilirubin (TBIL), estimated glomerular filtration rate (eGFR), uric acid (UA) and D-Dimer (DD). All these blood markers data are obtained by drawing blood samples from the anterior elbow vein by trained nursing staff after the patient is admitted to the hospital and sent to the hospital’s standard laboratory for testing.

### 2.5 Statistical analysis

All statistical analyses were performed using SPSS 26.0 and R 4.1.3. Continuous variables that conformed to normal distribution were described as mean ± standard deviation, and differences between groups were tested using the independent samples t-test. Continuous variables that did not conform to normal distribution were described as median (quartile), and differences between groups were analyzed using the non-parametric tests. Categorical variables were described as frequencies (percentages), and differences between groups were analyzed using the chi-square test. Univariate Cox regression analysis was used to evaluate the association between all variables and all-cause mortality of AECOPD, and then variables with *p* < 0.05 were selected for multivariate Cox regression analysis to evaluate the independent correlation between muscle mass related indicators and the risk of all-cause mortality. Then, based on gender (male or female), age (≤ 75 or > 75 years), BMI (< 24 or ≥ 24 kg/m^2^), smoking (yes or no), alcohol consumption (yes or no), dyslipidaemia (yes or no), pulmonary hypertension (absent, mild, moderate and severe), respiratory failure (yes or no), hypertension (yes or no), and respiratory failure severity (mild, moderate, severe), multiple subgroups were further assessed for stratified associations of muscle mass with all-cause mortality. The robustness of the association between muscle mass and all-cause mortality was subsequently revalidated in sensitivity analyses excluding patients with diabetes and pulmonary heart disease. Finally, receiver operating characteristic curve (ROC) and restricted cubic spline (RCS) plot were used to verify the predictive value of muscle mass for all-cause mortality and the potential nonlinear association with all-cause mortality. All tests were two-sided, and *p* values of less than 0.05 were considered to indicate statistical significance.

## 3 Results

### 3.1 Baseline characteristics

A total of 319 patients were enrolled in this study, with a median follow-up time of 14.63 (6.33, 21.13) months, and a total of 113 (35.4%) patients experienced all-cause mortality. As shown in Table 1, patients in the all-cause mortality group were significantly older and had a higher prevalence of dyslipidaemia and respiratory failure (*p* < 0.05). In addition, intubation time, heart rate, lactate level, PCT, AST, DD and mean intermuscular fat density were significantly higher in the all-cause mortality group, while SBP, DBP, serum calcium, ALB, and eGFR were significantly lower (*p* < 0.05).

**Table 1:** Baseline characteristics according to all-cause mortality.

Variables	Total population	None-all-cause mortality	All-cause mortality	*p*-value
*N*	319	206	113	
Age, years	75 (68, 81)	73 (67, 80)	79 (71, 83)	< 0.001
Gender, *n* (%)				0.951
Female	52 (16.30%)	34 (16.50%)	18 (15.93%)	
Male	267 (83.70%)	172 (83.50%)	95 (84.07%)	
Smoking history, *n* (%)	199 (62.38%)	128 (62.14%)	71 (62.83%)	0.963
Alcohol intake, *n* (%)	102 (31.97%)	64 (31.07%)	38 (33.63%)	0.464
Diabetes, *n* (%)	54 (16.93%)	35 (16.99%)	19 (16.81%)	0.862
Hypertension, *n* (%)	143 (44.83%)	97 (47.09%)	46 (40.71%)	0.229
Dyslipidaemia, *n* (%)	153 (48.0)	88 (42.7)	65 (57.5)	0.018
Pulmonary heart disease, *n* (%)	57 (17.87%)	33 (16.02%)	24 (21.24%)	0.229
Pulmonary artery pressure, mmHg	44 (35, 57)	43 (35, 56)	44 (37, 59)	0.416
Pulmonary hypertension, *n* (%)				0.612
No	108 (35.53%)	74 (37.56%)	34 (31.78%)	
Mild	85 (27.96%)	51 (25.89%)	34 (31.78%)	
Moderate	80 (26.32%)	52 (26.40%)	28 (26.17%)	
Severe	31 (10.20%)	20 (10.15%)	11 (10.28%)	
Respiratory failure severity, *n* (%)				0.411
Mild	129 (42.57%)	87 (44.85%)	42 (38.53%)	
Moderate	51 (16.83%)	30 (15.46%)	21 (19.27%)	
Severe	123 (40.59%)	77 (39.69%)	46 (42.20%)	
Respiratory failure, *n* (%)	192 (60.19%)	112 (54.37%)	80 (70.80%)	0.002
Heart failure, *n* (%)	40 (12.54%)	23 (11.17%)	17 (15.04%)	0.192
Duration of intubation, hours	0.90 ± 3.41	0.31 ± 1.72	1.96 ± 5.08	0.001
BMI, kg/m^2^	20.8 (18.3, 23.2)	20.9 (18.6, 23.1)	20.2 (17.9, 23.4)	0.441
SBP, mmHg	134 ± 22	136 ± 22	131 ± 21	0.029
DBP, mmHg	77 ± 14	79 ± 14	75 ± 14	0.014
HR, bpm	93 (82, 105)	91 (80, 101)	96 (85, 110)	0.003
PH	7.39 (7.32, 7.42)	7.38 (7.32, 7.42)	7.40 (7.33, 7.43)	0.304
FiO_2_	0.21 (0.21, 0.30)	0.21 (0.21, 0.30)	0.21 (0.21, 0.33)	0.052
SaO_2_, (%)	96.3 (92.4, 98.4)	96.2 (92.2, 98.3)	96.4 (92.9, 98.5)	0.583
LAC, mmol/L	1.40 (1.00, 1.90)	1.30 (1.00, 1.80)	1.50 (1.00, 2.00)	0.025
Sodium, mmol/L	139.0 (137.0, 142.0)	139.0 (136.0, 141.0)	140.0 (137.0, 142.0)	0.081
Potassium, mmol/L	3.90 ± 0.60	3.88 ± 0.57	3.94 ± 0.65	0.397
Calcium, mmol/L	2.03 (1.14, 2.22)	2.05 (1.15, 2.24)	1.28 (1.13, 2.20)	0.118
GLU, mmol/L	7.3 (6.1, 9.8)	7.1 (6.1, 9.5)	7.5 (6.2, 10.2)	0.136
CRP, mg/L	23 (7, 81)	20 (7, 74)	26 (10, 94)	0.170
PCT, ng/mL	0.10 (0.05, 0.30)	0.09 (0.05, 0.28)	0.14 (0.06, 0.30)	0.029
WBC, x10^9^/L	8.7 (6.7, 12.0)	8.2 (6.3, 11.1)	9.8 (7.6, 13.2)	0.004
Hb, g/L	131 (116, 146)	133 (118, 148)	124 (112, 142)	0.022
PLT, x10^9^/L	180 (115, 241)	184 (132, 243)	157 (28, 235)	0.010
ALB, g/L	33.2 ± 4.5	34.0 ± 4.4	31.8 ± 4.4	<0.001
ALT, U/L	17 (12, 28)	17 (12, 28)	18 (12, 26)	0.996
AST, U/L	24 (18, 32)	23 (17, 30)	25 (19, 35)	0.043
TBIL, μmol/L	9.0 (7.0, 13.0)	9.0 (7.0, 12.0)	10.0 (8.0, 13.0)	0.415
eGFR, ml/min/1.73m^2^	92 (71, 102)	94 (78, 103)	88 (56, 101)	0.010
UA, μmol/L	293 (212, 382)	293 (220, 367)	298 (194, 422)	0.655
DD, mg/L	0.87 (0.42, 1.53)	0.68 (0.33, 1.30)	1.29 (0.69, 2.38)	< 0.001
CSA, cm^2^	72.14 ± 19.21	75.35 ± 18.36	66.28 ± 19.42	< 0.001
SMI, cm^2^/m^2^	26.08 (22.39, 30.30)	27.13 (24.29, 31.06)	23.84 (20.59, 28.79)	< 0.001
Standardized SMI	−0.12 (−0.58, 0.40)	0.01 (−0.35, 0.50)	−0.40 (−0.81, 0.22)	< 0.001
SMI subgroup				< 0.001
Low SMI group	160 (50.2)	85 (41.3)	75 (66.4)	
High SMI group	159 (49.8)	121 (58.7)	38 (33.6)	
IMAT, cm^2^	6.66 (3.18, 11.89)	6.73 (3.44, 11.89)	6.34 (2.65, 12.11)	0.639
Mean muscle density, HU	27.40 ± 8.28	27.65 ± 7.87	26.94 ± 8.99	0.463
Mean intermuscular fat density, HU	−54.99 ± 7.27	−55.80 ± 7.19	−53.51 ± 7.21	0.007

BMI, body mass index; SBP, systolic blood pressure; DBP, diastolic blood pressure; HR, heart rate; FiO_2_, fraction of inspired oxygen; SaO_2_, arterial oxygen saturation; LAC, lactate; GLU, glucose; CRP, C-reactive protein; PCT, procalcitonin; WBC, white blood cell; Hb, hemoglobin; PLT, platele; ALB, albumin; ALT, alanine aminotransferase; AST, aspartate aminotransferase; TBIL, total bilirubin; eGFR, estimated glomerular filtration rate; UA, uric acid; DD, D-Dimer; SMI, skeletal muscle index; IMAT, intermuscular fat; CSA, muscle cross-sectional area.

As shown in Table 2, patients in the high SMI group exhibited higher BMI, calcium, SBP, hemoglobin and lower incidence of dyslipidaemia compared to the low SMI group (*p* < 0.05). In contrast, heart rate, CRP, PCT and WBC were lower in the high SMI group (*p* < 0.05). Specifically, all-cause mortality was significantly lower in the high SMI group than in the low SMI group (*p* < 0.001).

**Table 2:** Baseline characteristics of subgroups according to median SMI.

Variables	Low SMI group	High SMI group	*p*-value
Age, years	75.34 ± 9.18	73.74 ± 9.45	0.126
Gender, *n* (%)			0.376
Female	29 (18.1)	23 (14.5)	
Male	131 (81.9)	136 (85.5)	
Smoking history, *n* (%)	100 (62.5)	99 (62.3)	0.965
Alcohol intake, *n* (%)	49 (30.6)	53 (33.3)	0.604
Diabetes, *n* (%)	24 (15.0)	30 (18.9)	0.357
Hypertension, *n* (%)	59 (36.9)	84 (52.8)	0.004
Dyslipidaemia, *n* (%)	87 (54.4)	66 (41.5)	0.021
Pulmonary heart disease, *n* (%)	31 (19.4)	26 (16.4)	0.481
Pulmonary artery pressure, mmHg	44.00 (37.00, 58.00)	42.00 (34.00, 56.50)	0.109
Pulmonary hypertension, *n* (%)			0.375
No	47 (30.9)	61 (40.1)	
Mild	47 (30.9)	38 (25.0)	
Moderate	41 (27.0)	39 (25.7)	
Severe	17 (11.2)	14 (9.2)	
Respiratory failure severity, *n* (%)			0.701
Mild	65 (41.7)	64 (43.5)	
Moderate	29 (18.6)	22 (15.0)	
Severe	62 (39.7)	61 (41.5)	
Respiratory failure, *n* (%)	102 (63.7)	90 (56.6)	0.192
Heart failure, *n* (%)	22 (13.8)	18 (11.3)	0.512
Duration of intubation, hours	1.16 ± 3.50	0.64 ± 3.30	0.172
BMI, kg/m^2^	19.52 ± 3.18	22.65 ± 3.93	< 0.001
SBP, mmHg	131.20 ± 21.89	137.31 ± 21.19	0.012
DBP, mmHg	76.32 ± 14.40	78.41 ± 14.12	0.192
HR, bpm	99.49 ± 18.19	89.03 ± 16.58	< 0.001
PH	7.37 ± 0.09	7.36 ± 0.08	0.176
FiO_2_	0.26 ± 0.09	0.26 ± 0.07	0.489
SaO_2_, (%)	94.70 ± 5.87	93.63 ± 8.38	0.189
LAC, mmol/L	1.30 (0.90, 1.80)	1.30 (0.95, 1.80)	0.939
Sodium, mmol/L	139.39 ± 4.84	138.62 ± 4.63	0.150
Potassium, mmol/L	3.89 ± 0.56	3.92 ± 0.64	0.134
Calcium, mmol/L	1.68 ± 0.53	1.77 ± 0.55	0.039
GLU, mmol/L	7.30 (6.00, 9.60)	7.20 (6.10, 9.50)	0.583
CRP, mg/L	36.50 (9.40, 114.00)	19.40 (5.50, 50.05)	0.005
PCT, ng/mL	0.12 (0.06, 0.31)	0.08 (0.05, 0.22)	0.019
WBC, x10^9^/L	9.79 (6.78, 13.41)	7.75 (6.42, 10.41)	0.004
Hb, g/L	127.36 ± 24.02	133.79 ± 23.52	0.016
PLT, x10^9^/L	167.00 (37.00, 240.00)	182.00 (134.50, 237.50)	0.042
ALB, g/L	32.23 ± 4.74	34.23 ± 3.98	< 0.001
ALT, U/L	16.00 (11.00, 26.00)	17.00 (12.00, 27.50)	0.800
AST, U/L	23.00 (18.00, 32.00)	23.00 (18.00, 30.00)	0.188
TBIL, μmol/L	10.00 (8.00, 13.00)	10.00 (7.00, 13.00)	0.627
eGFR, ml/min/1.73m^2^	92.20 (75.40, 103.40)	89.80 (67.80, 101.00)	0.186
UA, μmol/L	273.00 (199.00, 362.00)	313.00 (231.00, 385.00)	0.019
DD, mg/L	1.05 (0.47, 1.81)	0.68 (0.37, 1.44)	0.001
CSA, cm^2^	58.68 ± 10.94	85.68 ± 15.94	< 0.001
SMI, cm^2^/m^2^	22.45 (19.48, 24.48)	30.41 (28.11, 35.38)	< 0.001
Standardized SMI	−0.58 (−0.95, −0.32)	0.42 (0.13, 1.04)	< 0.001
IMAT, cm^2^	5.54 (2.57, 11.31)	7.71 (3.69, 12.76)	0.043
Mean muscle density, HU	26.72 ± 8.67	28.08 ± 7.83	0.144
Mean intermuscular fat density, HU	−54.59 ± 7.74	−55.39 ± 6.76	0.326
All-cause mortality			< 0.001
Yes	75 (46.9)	38 (23.9)	
No	85 (53.1)	121 (76.1)	

BMI, body mass index; SBP, systolic blood pressure; DBP, diastolic blood pressure; HR, heart rate; FiO_2_, fraction of inspired oxygen; SaO_2_, arterial oxygen saturation; LAC, lactate; GLU, glucose; CRP, C-reactive protein; PCT, procalcitonin; WBC, white blood cell; Hb, hemoglobin; PLT, platele; ALB, albumin; ALT, alanine aminotransferase; AST, aspartate aminotransferase; TBIL, total bilirubin; eGFR, estimated glomerular filtration rate; UA, uric acid; DD, D-Dimer; SMI, skeletal muscle index; IMAT, intermuscular fat; CSA, muscle cross-sectional area.

### 3.2 Predictive value of SMl for all-cause mortality and its nonlinear association with all-cause mortality

**Figure 2:**
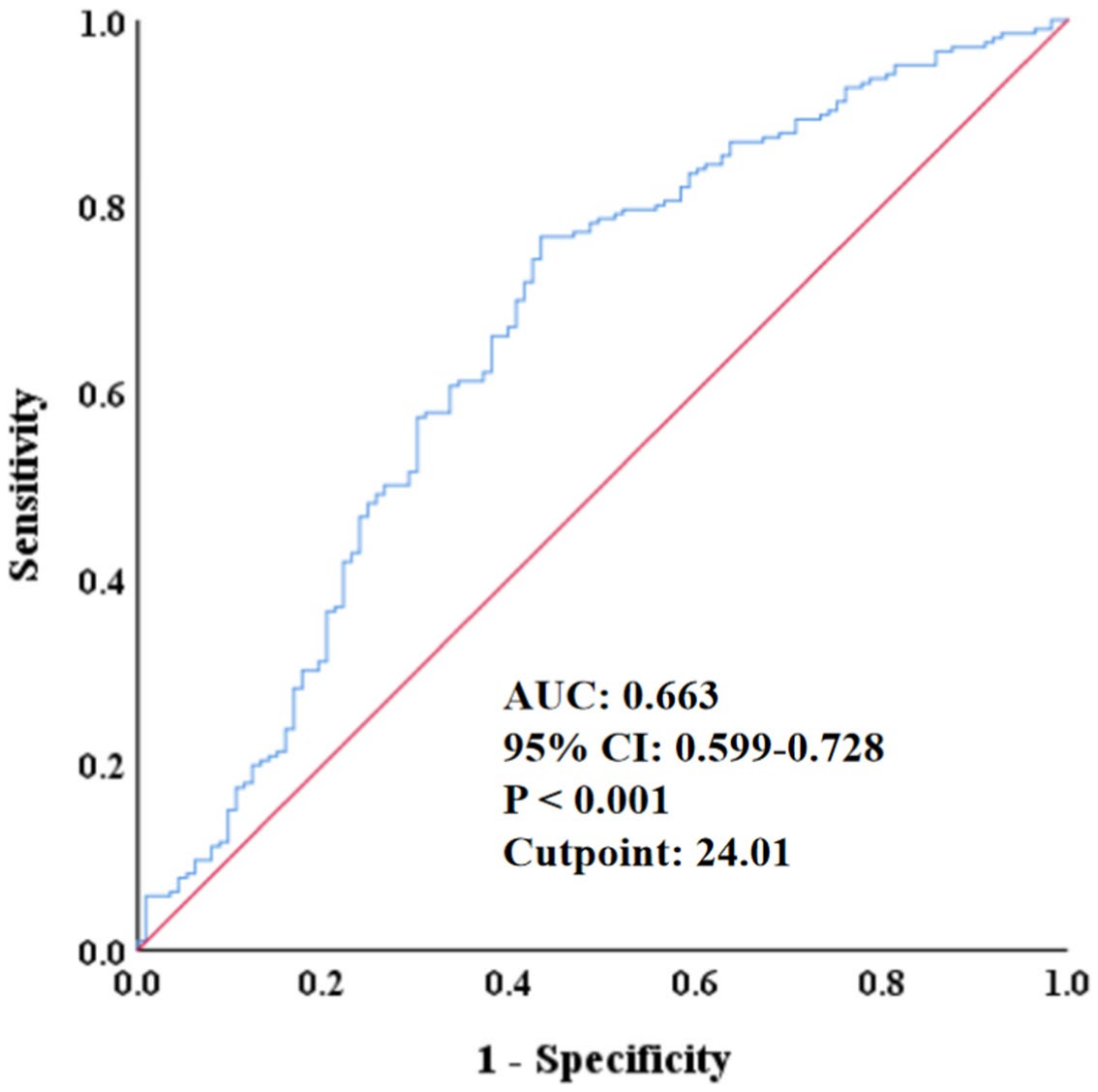
ROC curve of the predictive value of SMI for all-cause mortality. ROC, receiver operating characteristic; AUC, area under the curve; CI, confidence interval; SMI, skeletal muscle index.

**Figure 3:**
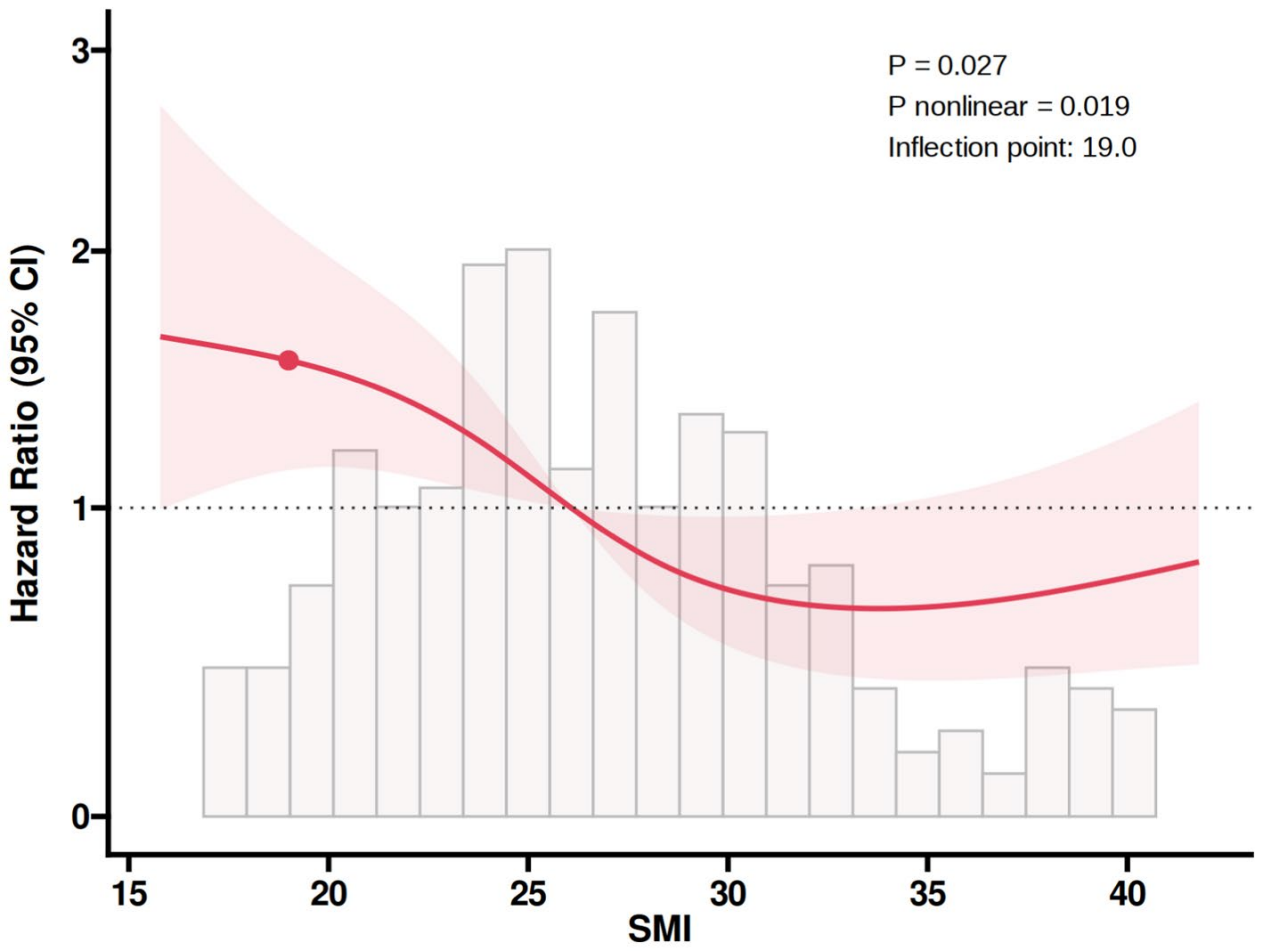
RCS plot of the non-linear association of SMI with all-cause mortality. RCS, restricted cubic spline; SMI, skeletal muscle index.

The ROC analysis in Figure 2 showed that the area under the curve of SMI in predicting all-cause mortality was 0.663 (95% CI: 0.559–0.728, *p* < 0.001), with a cut-off point of 24.01, a sensitivity of 56.6%, a specificity of 76.7%, 48 false positives, and a true positive rate of 20.06%, indicating that SMI had certain predictive ability. In addition, the RCS regression model analysis showed that the relationship between SMI and all-cause mortality was statistically significant (*p* = 0.027), and the *p* value of the non-linear part was 0.019, further validating the non-linear character of the relationship (Figure 3).

### 3.3 Kaplan–Meier survival curves stratified by SMI levels

**Figure 4:**
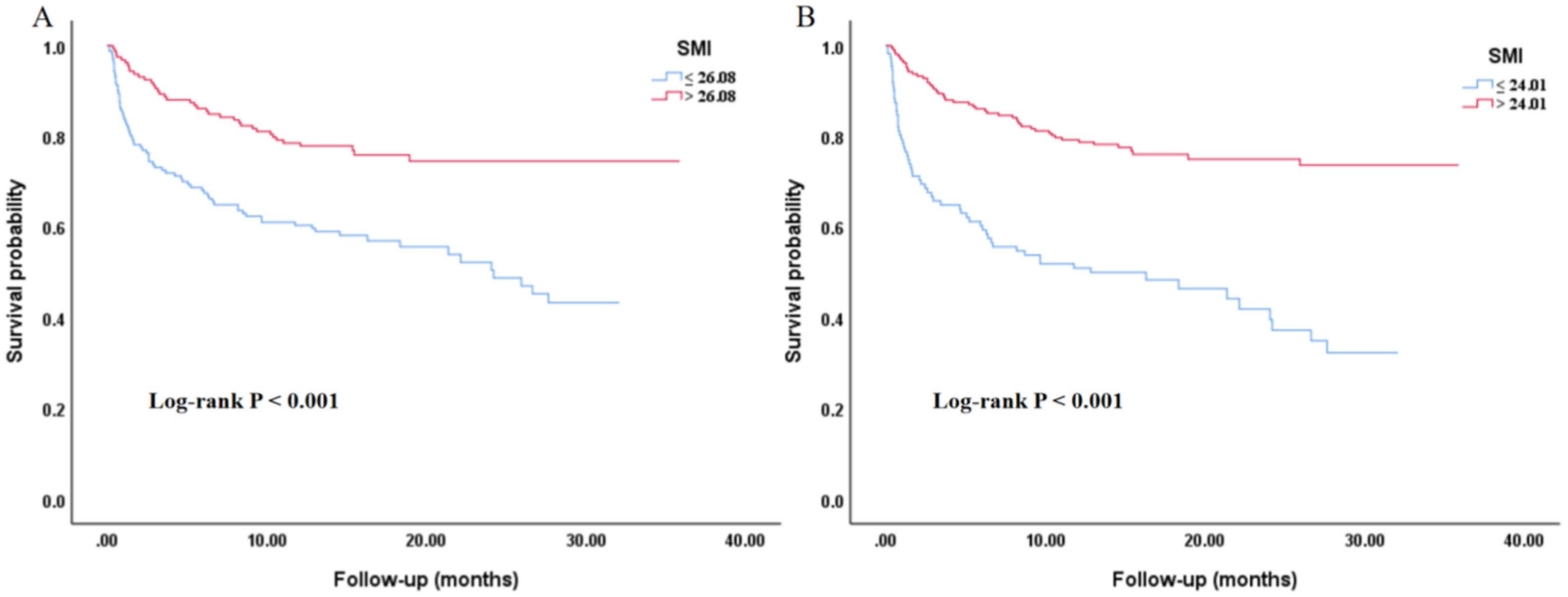
Kaplan–Meier curves of the SMI with all-cause mortality. **(A)** Grouped by the median value of SMI. **(B)** Grouped by the cut-off point of SMI. SMI, skeletal muscle index.

The Kaplan–Meier survival curves analysis in Figure 4 indicated that regardless of whether SMI was grouped by median (A) or by cut-off point (B), there were significant differences in the survival probability of all-cause mortality among different SMI groups, with the low SMI group having a poorer prognosis (*p* < 0.001).

### 3.4 Association between SMI and all-cause mortality

Table 3 showed the results of the univariate Cox regression analysis for all-cause mortality. The results showed that age, dyslipidaemia, respiratory failure, duration of intubation, SBP, DPB, heart rate, FiO2, lactate, sodium, calcium, ALB, ALT, AST, eGFR, DD, CSA, SMI, standardized SMI, SMI subgroups (grouped by median), SMI subgroup (grouped by cut-off point) and mean intermuscular fat density were significantly associated with all-cause mortality (*p* < 0.05). Multivariate Cox regression analysis (Table 4) showed that SMI, standardized SMI, CSA, and mean intermuscular fat density were significantly associated with all-cause mortality in both models 1 and 2 (*p* < 0.05). In fully adjusted model 3, regardless of whether SMI was grouped based on the median of 26.08 or the cut-off point of 24.01, the low SMI group had a higher risk of all-cause mortality (HR: 0.495, 95% CI: 0.330–0.743, *p* = 0.001; HR: 0.400, 95% CI: 0.270–0.592, *p* < 0.001). Moreover, as a continuous variable, lower levels of SMI were independently associated with a higher risk of all-cause mortality (SMI, HR = 0.964, 95% CI: 0.934–0.996, *p* = 0.027; Standardized SMI, HR = 0.748, 95% CI: 0.578–0.967, *p* = 0.027).

**Table 3:** Univariate Cox regression analysis of all-cause mortality.

Variables	HR	95% CI	*p*-value
Age	1.039	1.017–1.061	< 0.001
Gender			
Female	Ref		
Male	1.040	0.628–1.722	0.878
Smoking history	1.088	0.743–1.595	0.664
Alcohol intake	1.116	0.755–1.648	0.583
Diabetes	1.009	0.616–1.552	0.971
Hypertension	0.754	0.518–1.098	0.141
Dyslipidaemia	1.662	1.144–2.413	0.008
Pulmonary heart disease	1.257	0.800–1.976	0.322
Pulmonary artery pressure	1.007	0.995–1.019	0.227
Pulmonary hypertension			
No	Ref		
Mild	1.347	0.837–2.168	0.173
Moderate	1.220	0.740–2.013	0.436
Severe	1.365	0.692–2.695	0.370
Respiratory failure severity			
Mild	Ref		
Moderate	1.322	0.782–2.232	0.297
Severe	1.316	0.864–2.004	0.201
Respiratory failure	1.931	1.286–2.900	0.002
Heart failure	1.535	0.915–2.574	0.104
Duration of intubation	1.068	1.038–1.099	< 0.001
BMI	0.973	0.926–1.022	0.270
SBP	0.990	0.981–0.998	0.019
DBP	0.984	0.971–0.997	0.016
Heart rate	1.017	1.007–1.027	0.001
PH value	1.148	0.117–11.260	0.906
FiO_2_	15.456	1.862–128.271	0.011
SaO_2_	1.011	0.985–1.038	0.393
LAC	1.260	1.076–1.475	0.004
Sodium	1.044	1.003–1.086	0.036
Potassium	1.240	0.902–1.704	0.185
Calcium	0.701	0.498–0.987	0.042
GLU	1.042	0.999–1.088	0.059
CRP	1.001	0.999–1.003	0.418
PCT	0.968	0.861–1.088	0.587
WBC	1.028	1.000–1.056	0.052
Hb	0.992	0.984–1.000	0.054
PLT	1.000	0.999–1.002	0.632
ALB	0.910	0.873–0.948	< 0.001
ALT	1.002	1.001–1.003	0.001
AST	1.001	1.000–1.001	0.005
TBIL	1.018	0.991–1.046	0.194
eGFR	0.987	0.981–0.994	< 0.001
UA	1.001	1.000–1.002	0.178
DD	1.141	1.095–1.190	< 0.001
CSA	0.975	0.965–0.986	< 0.001
SMI	0.931	0.904–0.960	< 0.001
Standardized SMI	0.566	0.444–0.720	< 0.001
SMI subgroup (26.08)			
Low SMI group	Ref		
High SMI group	0.415	0.281–0.613	< 0.001
SMI subgroup (24.01)			
Low SMI group	Ref		
High SMI group	0.313	0.216–0.454	< 0.001
IMAT	0.994	0.967–1.021	0.641
Mean muscle density	0.992	0.970–1.015	0.512
Mean intermuscular fat density	1.043	1.016–1.070	0.002

BMI, body mass index; SBP, systolic blood pressure; DBP, diastolic blood pressure; FiO_2_, fraction of inspired oxygen; SaO_2_, arterial oxygen saturation; LAC, lactate; GLU, glucose; CRP, C-reactive protein; PCT, procalcitonin; WBC, white blood cell; Hb, hemoglobin; PLT, platele; ALB, albumin; ALT, alanine aminotransferase; AST, aspartate aminotransferase; TBIL, total bilirubin; eGFR, estimated glomerular filtration rate; UA, uric acid; DD, D-Dimer; SMI, skeletal muscle index; IMAT, intermuscular fat; CSA, muscle cross-sectional area; HR, hazard ratio; CI, confidence interval.

**Table 4:** Multivariate association of skeletal muscle index with all-cause mortality.

Variables	Model 1			Model 2			Model 3		
	HR	95% CI	*p*-value	HR	95% CI	*p*-value	HR	95% CI	*p*-value
Low SMI group (≤ 26.08)	Ref			Ref			Ref		
High SMI group (> 26.08)	0.433	0.292–0.640	< 0.001	0.531	0.353–0.798	0.002	0.495	0.330–0.743	0.001
Low SMI group (≤ 24.01)	Ref			Ref			Ref		
High SMI group (> 24.01)	0.329	0.227–0.478	< 0.001	0.427	0.289–0.630	< 0.001	0.400	0.270–0.592	< 0.001
SMI	0.933	0.905–0.962	< 0.001	0.960	0.931–0.990	0.009	0.964	0.934–0.996	0.027
Standardized SMI	0.574	0.448–0.735	< 0.001	0.720	0.563–0.920	0.009	0.748	0.578–0.967	0.027
CSA	0.977	0.966–0.988	< 0.001	0.989	0.978–1.000	0.060	0.992	0.980–1.004	0.169
Intermuscular fat area	0.984	0.957–1.012	0.256	0.986	0.957–1.016	0.349	0.998	0.970–1.028	0.909
Average muscle density	1.004	0.980–1.029	0.728	1.014	0.989–1.039	0.276	1.004	0.981–1.029	0.715
Mean intermuscular fat density	1.050	1.023–1.078	< 0.001	1.032	1.004–1.060	0.023	1.104	0.986–1.044	0.325

Model 1: adjusted for age; Model 2: adjusted for age, dyslipidaemia, respiratory failure, heart rate, SBP, DBP, and length of intubation; Model 3: adjusted for Model 2 + DD, eGFR, ALB, FiO_2_, ALT, AST, serum calcium, serum sodium, and LAC. SBP, systolic blood pressure; DBP, diastolic blood pressure; DD, D-Dimer; eGFR, estimated glomerular filtration rate; ALB, albumin; FiO_2_, fraction of inspired oxygen; ALT, alanine aminotransferase; AST, aspartate aminotransferase; SMI, skeletal muscle index; HR, hazard ratio; CI, confidence interval.

### 3.5 Stratified association of SMI with all-cause mortality

In Table 5, subgroup analysis showed that the risk of all-cause mortality in the high SMl group (grouped by median) was lower than that in the low SMl group (grouped by median) in the subgroups of male, smoking, alcohol intake, dyslipidemia, moderate-to-severe pulmonary hypertension, respiratory failure, no hypertension, and moderate-to-severe respiratory failure. However, in the female subgroup, the risk of all-cause mortality in the high SMl group was significantly higher than that in the low SMl group, with a HR of 65.448 (95% CI: 3.148–1360.518) (*p* = 0.007). Besides, when grouped according to the cut-off point, compared with the high SMI group, the low SMI group also had a higher risk of all-cause mortality in the subgroups of male, smoking, non-smoking, alcohol intake, no alcohol intake, dyslipidemia, moderate-to-severe pulmonary hypertension, respiratory failure, hypertension, no hypertension, and mild to moderate-to-severe respiratory failure (*p* < 0.05). In addition, in the subgroups of males, > 75 years of age, alcohol consumption, dyslipidaemia, moderate-to-severe pulmonary hypertension, respiratory failure, no hypertension, and moderate-to-severe respiratory failure, elevated SMI and standardized SMI were correlated with reduced all-cause mortality (*p* < 0.05).

**Table 5:** Multivariate stratified association between skeletal muscle index and all-cause mortality.

Subgroups	High SMI group vs. Low SMI group (26.08)			High SMI group vs. Low SMI group (24.01)			SMI			Standardized SMI		
	HR	95% CI	*p*-value	HR	95% CI	*p*-value	HR	95% CI	*p*-value	HR	95% CI	*p*-value
Gender												
Male	0.391	0.252–0.606	< 0.001	0.312	0.206–0.474	< 0.001	0.932	0.898–0.967	< 0.001	0.569	0.423–0.766	< 0.001
Female	65.448	3.148–1360.518	0.007	1.903	0.264–13.695	0.523	1.123	0.971–1.300	0.119	2.537	0.788–8.164	0.119
Age												
≤ 75	0.603	0.292–1.244	0.171	0.293	0.155–0.556	< 0.001	0.978	0.933–1.025	0.348	0.836	0.576–1.215	0.348
> 75	0.587	0.315–1.092	0.093	0.394	0.240–0.648	< 0.001	0.948	0.908–0.990	0.015	0.653	0.463–0.920	0.015
Smoking												
Yes	0.350	0.211–0.581	< 0.001	0.356	0.218–0.579	< 0.001	0.978	0.939–1.019	0.294	0.838	0.603–1.165	0.294
No	0.637	0.278–1.460	0.286	0.300	0.152–0.591	< 0.001	0.937	0.877–1.001	0.054	0.595	0.351–1.009	0.054
Alcohol intake												
Yes	0.365	0.183–0.727	0.004	0.367	0.190–0.707	0.003	0.928	0.883–0.976	0.004	0.552	0.368–0.826	0.004
No	0.647	0.377–1.110	0.114	0.357	0.219–0.581	< 0.001	0.977	0.937–1.019	0.277	0.830	0.594–1.161	0.277
Dyslipidemia												
Yes	0.515	0.284–0.934	0.029	0.375	0.217–0.649	< 0.001	0.954	0.915–0.995	0.029	0.688	0.492–0.962	0.029
No	0.573	0.279–1.178	0.130	0.533	0.260–1.089	0.084	0.964	0.905–1.027	0.257	0.745	0.448–1.239	0.257
Pulmonary arterial hypertension												
None-mild	0.763	0.425–1.370	0.364	0.554	0.304–1.010	0.054	0.983	0.941–1.027	0.439	0.870	0.613–1.237	0.439
Moderate–severe	0.267	0.125–0.569	0.001	0.219	0.108–0.442	< 0.001	0.955	0.915–0.997	0.036	0.694	0.493–0.976	0.036
Respiratory failure												
Yes	0.336	0.202–0.560	< 0.001	0.316	0.193–0.518	< 0.001	0.936	0.903–0.971	< 0.001	0.590	0.443–0.787	< 0.001
No	0.914	0.406–2.058	0.828	0.763	0.305–1.909	0.563	1.020	0.948–1.098	0.588	1.176	0.653–2.117	0.588
Hypertension												
Yes	0.788	0.387–1.604	0.511	0.344	0.185–0.639	0.001	0.976	0.928–1.026	0.340	0.823	0.552–1.228	0.340
No	0.331	0.186–0.588	< 0.001	0.333	0.197–0.562	< 0.001	0.936	0.898–0.975	0.001	0.588	0.424–0.816	0.001
Respiratory failure severity												
Mild	0.723	0.317–1.649	0.441	0.373	0.200–0.695	0.002	0.993	0.925–1.066	0.846	0.945	0.537–1.664	0.846
Moderate–severe	0.324	0.186–0.563	< 0.001	0.277	0.165–0.466	< 0.001	0.931	0.896–0.966	<0.001	0.562	0.416–0.759	< 0.001

SMI, skeletal muscle index; HR, hazard ratio; CI, confidence interval.

### 3.6 Sensitivity analysis of SMI and all-cause mortality

Sensitivity analyses showed (Table 6) that, after excluding patients with diabetes, regardless of whether SMI was grouped based on the median of 26.08 or the cut-off point of 24.01, the low SMI group had a higher risk of all-cause mortality (HR: 0.524, 95% CI: 0.337–0.815, *p* = 0.004; HR: 0.418, 95% CI: 0.276–0.635, *p* < 0.001). While the association between SMl and all-cause mortality remained significant when patients pulmonary heart disease were excluded. In all-adjusted model 3, the risk of high SMl group was lower than that of low SMI group (HR: 0.471, 95% CI: 0.298–0.744, *p* = 0.001; HR: 0.376, 95% CI: 0.244–0.579, *p* < 0.001). And increased SMI and standardized SMI were linked to a reduction in all-cause mortality (95% CI: 0.944–0.978, *p* = 0.002; 95% CI: 0.472–0.839, *p* = 0.002).

**Table 6:** Multivariate association of skeletal muscle index with all-cause mortality: patients with diabetes or pulmonary heart disease were excluded.

Variables	Model 1			Model 2			Model 3		
	HR	95% CI	*p*-value	HR	95% CI	*p*-value	HR	95% CI	*p*-value
Exclusion of people with diabetes									
Low SMI group (≤ 26.08)	Ref			Ref			Ref		
High SMI group (> 26.08)	0.470	0.307–0.721	0.001	0.553	0.356–0.858	0.008	0.524	0.337–0.815	0.004
Low SMI group (≤ 24.01)	Ref			Ref			Ref		
High SMI group (> 24.01)	0.377	0.251–0.567	< 0.001	0.477	0.314–0.725	0.001	0.418	0.276–0.635	< 0.001
SMI	0.943	0.911–0.976	0.001	0.974	0.941–1.007	0.119	0.979	0.944–1.016	0.262
Standardized SMI	0.625	0.474–0.823	0.001	0.807	0.617–1.057	0.119	0.845	0.629–1.135	0.262
Exclusion of patients with pulmonary heart disease									
Low SMI group (≤ 26.08)	Ref			Ref			Ref		
High SMI group (> 26.08)	0.405	0.261–0.630	< 0.001	0.527	0.331–0.840	0.007	0.471	0.298–0.744	0.001
Low SMI group (≤ 24.01)	Ref			Ref			Ref		
High SMI group (> 24.01)	0.308	0.203–0.470	< 0.001	0.362	0.236–0.555	< 0.001	0.376	0.244–0.579	< 0.001
SMI	0.916	0.884–0.949	< 0.001	0.938	0.906–0.972	<0.001	0.944	0.911–0.978	0.002
Standardized SMI	0.496	0.374–0.658	0.003	0.601	0.453–0.797	<0.001	0.630	0.472–0.839	0.002

SMI, skeletal muscle index; HR, hazard ratio; CI, confidence interval.

## 4 Discussion

This study investigated the association between skeletal muscle mass and all-cause mortality risk in patients with AECOPD. After stepwise adjustment for all covariates, higher levels of SMI were independently associated with lower risk of all-cause mortality. These associations were further validated in multiple subgroup analyses and remained significant even after excluding patients with diabetes or pulmonary heart disease. Additionally, ROC not only validated the predictive value of SMI for all-cause mortality, but the RCS also revealed a potential nonlinear association between them. Finally, Kaplan–Meier survival curves analysis showed significant differences in survival probability between SMI groups, regardless of whether SMI was grouped by median or cut-off values, with the low SMI group exhibiting poorer prognosis. These findings highlight the certain diagnostic performance of SMI for all-cause mortality in AECOPD patients, underscoring the need for routine SMI screening and early intervention to improve risk monitoring, prognostic management, and reduce premature mortality in this population. Current evidence shows that muscle mass plays an important role in the occurrence and development of COPD, especially SMI and sarcopenia. However, there are few studies on SMI in COPD patients, and sarcopenia has received more attention. For example, an observational study in elderly COPD patients found that 25% had comorbid malnutrition, while sarcopenia prevalence was as high as 1 in 7 (30). Nearly 10% of individuals suffer from dystrophy-sarcopenia syndrome, which will greatly promote the occurrence and development of COPD, leading to aggravation of the disease and even poor prognosis (30). Secondly, an epidemiological survey involving 622 patients with stable COPD reported that up to 15% of patients with stable COPD were suffering from sarcopenia and were further reduced in motor function, quality of life and health status (14). In addition, in a small sample cross-sectional study of only 91 COPD patients, the proportion of sarcopenia in the total population was as high as 39.6%, and the percentage of body fat, BMl, and total lean mass were even lower. More importantly, sarcopenia has also been confirmed to be closely related to worse motor function in COPD patients, and is independent of traditional COPD risk factors (9). Furthermore, in a systematic review and meta-analysis that including 23 cross-sectional and cohort studies and 9,637 individuals over the age of 40, Sepúlveda-Loyola et al. found that 15.5–34% of participants developed sarcopenia, with no significant gender differences (16). Although they did not confirm the association between oxidative stress or inflammatory factor levels and sarcopenia, they came to a very important conclusion: patients with sarcopenia often have worse quality of life, motor function, and even respiratory function, which indicates that timely diagnosis and intervention of muscle mass loss or sarcopenia play a crucial role in improving respiratory function, motor function, quality of life, and poor prognosis of COPD patients (16). Moreover, in a longitudinal cohort study, researchers utilized the pectoralis muscle area measured from chest CT scans as a quantitative indicator of muscle mass. The study found that as muscle mass decreased, the all-cause mortality rate among patients with COPD increased (31). Furthermore, a previous study have found that low muscle mass measured by chest CT scans was associated with increased mortality in current smokers without airflow obstruction (18). Similarly, in a small cross-sectional study conducted in a Chinese population, it was found that the index of muscle mass reduction was closely associated with the frequent exacerbation of respiratory symptoms in patients with AECOPD, particularly increasing the likelihood of respiratory failure and severe pneumonia (32). Furthermore, in a survey conducted among a South Korean population, it was discovered that the reduction in muscle mass was often associated with the exacerbation of COPD. However, in clinical practice, only a small proportion of individuals undergo assessments for muscle mass (33). In a small cohort study, researchers found that psoas muscle density (PsD) assessed by chest CT was significantly lower in COPD patients compared to healthy controls and independently associated with long-term all-cause mortality, suggesting that PsD, as an easily measurable indicator, may provide valuable prognostic information and improve risk stratification and treatment decisions in COPD patients (34). While these studies suggest a link between muscle mass loss and COPD severity, inconsistent findings may be due to variations in sample size and population heterogeneity. And to the best of our knowledge, the association between muscle mass loss and all-cause mortality in AECOPD remains unclear. Therefore, our findings not only once again support the conclusion that muscle mass loss is important in COPD, but also fill additional knowledge gaps, that is, this study revealed a significant association between muscle mass loss and all-cause mortality in AECOPD patients, and reconfirmed the robustness of these results in multiple subgroup analyses and sensitivity analyses. We also innovatively explored a potential nonlinear association, providing a stronger theoretical basis for COPD clinical management. Our findings reaffirmed the impact of muscle mass loss in COPD and filled knowledge gaps by demonstrating a significant association between muscle mass loss and all-cause mortality in AECOPD patients through subgroup and sensitivity analyses. Meanwhile, innovatively explored a potential non-linear association between them, which provide greater clinical value and theoretical basis for the clinical management of COPD patients. In addition, in this study, we also found that the association between SMI and all-cause mortality risk was significantly different by gender. Male patients in the high SMI group had a significantly lower risk of death than those in the low SMI group, whereas the association was less pronounced among female patients. Jones et al. noted that males generally have higher muscle mass and greater metabolic capacity, and therefore are better able to cope with the negative effects of the disease in the presence of a higher SMI (14). In contrast, female patients may face more complex health risks, especially when skeletal muscle mass is higher, and other underlying health problems may increase mortality risk. Gender differences may be strongly associated with physiological factors such as muscle distribution, hormone levels, and metabolic rate, suggesting that the potential moderating effect of sex on the relationship between skeletal muscle mass and the risk of mortality needs to be considered in clinical interventions.

Despite these significant findings, the underlying biological mechanisms remain unclear. After conducting a literature review, we found that muscle mass loss was particularly common in patients with COPD. Studies suggest that muscle mass loss exacerbates COPD through multiple mechanisms, particularly in the development and progression of acute exacerbations. Firstly, the systemic inflammatory response associated with muscle mass loss is a key factor in AECOPD. Muscle mass loss is closely associated with a chronic low-grade inflammatory state. These inflammatory factors exacerbate both muscle loss and airway inflammation, increasing the risk of acute exacerbations (35). In addition, muscle atrophy and loss of function make the respiratory muscle groups (e.g., diaphragm and intercostal muscles) less powerful in COPD patients, leading to further deterioration of respiratory function. Respiratory muscle weakness due to muscle mass loss impairs airway clearance, increasing the risk of obstruction and contributing to AECOPD (36). Secondly, hypoxaemia is a common concomitant symptom in patients with COPD, and it has been proved to be an important causative factor of muscle loss. Long-term hypoxia not only promotes muscle atrophy, but also aggravates respiratory muscle fatigue through insufficient oxygenation, which further contributes to the decline of activity endurance and the frequent occurrence of acute exacerbations (37). Finally, muscle mass loss is also strongly associated with malnutrition in COPD patients. Muscle mass loss is usually accompanied by inadequate protein and energy intake, leading to systemic malnutrition, which in turn affects muscle synthesis and repair (37). The poor nutritional status in COPD patients exacerbates muscle loss and decreased function of respiratory muscle groups, which in turn increases the risk of AECOPD (38). Therefore, improving the nutritional status of COPD patients, especially increasing protein and energy intake, may help to slow the progression of muscle mass loss and effectively reduce the frequency of acute exacerbations. In conclusion, muscle mass loss may contribute to acute exacerbations of COPD through systemic inflammation, respiratory muscle weakness, hypoxemia, and malnutrition, ultimately increasing mortality risk. Early intervention targeting these mechanisms to improve patients’ muscle mass and nutritional status may provide new strategies for the treatment of COPD, reduce the burden of acute exacerbations, and improve patients’ quality of life.

Despite its valuable findings, this study had several limitations. First, in this study, the sample size was relatively small and may lack sufficient statistical efficacy; further expansion of the sample size and more in-depth research are needed in the future to continue to explore the relationship between the two. Second, this study only included patients with AECOPD and did not evaluate patients in stable phase, so the results may not be comprehensive. In the future, further studies are needed to validate the association between them in different COPD populations. Third, although this study confirmed the importance of muscle mass loss in the prognosis of AECOPD patients through SMl, there is no recognized standard, so sarcopenia has not been further evaluated, which is the biggest limitation of this study. In the future, the study design will be improved, the data will be collected comprehensively, and the correlation between muscle mass loss related indicators and the poor prognosis of COPD patients will be systematically evaluated. Fourth, the other indicators of muscle mass loss have not been confirmed to be significantly associated with all-cause mortality in AECOPD patients, which may lead to inconsistent results due to small sample sizes or large heterogeneity of the study population. In the future, more adequate studies will be conducted to explore their associations again. Fifth, since the patients with AECOPD included in this study were mostly severe cases, pulmonary function tests were not conducted on these patients. This led to the absence of a large number of pulmonary function parameters in this study and the inability to interpolate them. Therefore, during the analysis, we had to delete these indicators and were unable to assess the impact of pulmonary function on the research results. In future studies, we will re-verify the stability of the results in some COPD patients with milder conditions and explore the influence of pulmonary function on the results. Finally, this study still inevitably missed some important confounders such as occupational exposure, environmental pollution, diet and genetic susceptibility. Despite these limitations, our study still provides some clues and theoretical basis for the role of muscle mass in the clinical management of COPD patients, and makes a contribution to promoting the integration of muscle mass into the routine clinical management of COPD in the future.

## 5 Conclusion

Skeletal muscle mass, especially SMI, was strongly associated with the risk of all-cause mortality in AECOPD patients, indicating that SMI, as a reliable muscle mass related imaging indicator, can be used for prognostic risk assessment and personalized management of AECOPD patients. Through early identification and intervention of muscle mass loss, clinicians are able to improve patient prognosis and optimize treatment options. Future studies should further explore the impact of skeletal muscle mass on the long-term prognosis of COPD patients and reveal the underlying mechanisms. In addition, further consideration of the moderating effects of gender, nutritional status and other clinical factors on the relationship between SMl and the risk of death will help to develop more personalized treatment strategies.

## Data Availability

The original contributions presented in the study are included in the article/supplementary material, further inquiries can be directed to the corresponding authors.
